# On the Perception of Religious Group Membership from Faces

**DOI:** 10.1371/journal.pone.0014241

**Published:** 2010-12-07

**Authors:** Nicholas O. Rule, James V. Garrett, Nalini Ambady

**Affiliations:** 1 Psychology Department, University of Toronto, Toronto, Ontario, Canada; 2 Psychology Department, Tufts University, Medford, Massachusetts, United States of America; University of Minnesota, United States of America

## Abstract

**Background:**

The study of social categorization has largely been confined to examining groups distinguished by perceptually obvious cues. Yet many ecologically important group distinctions are less clear, permitting insights into the general processes involved in person perception. Although religious group membership is thought to be perceptually ambiguous, folk beliefs suggest that Mormons and non-Mormons can be categorized from their appearance. We tested whether Mormons could be distinguished from non-Mormons and investigated the basis for this effect to gain insight to how subtle perceptual cues can support complex social categorizations.

**Methodology/Principal Findings:**

Participants categorized Mormons' and non-Mormons' faces or facial features according to their group membership. Individuals could distinguish between the two groups significantly better than chance guessing from their full faces and faces without hair, with eyes and mouth covered, without outer face shape, and inverted 180°; but not from isolated features (i.e., eyes, nose, or mouth). Perceivers' estimations of their accuracy did not match their actual accuracy. Exploration of the remaining features showed that Mormons and non-Mormons significantly differed in perceived health and that these perceptions were related to perceptions of skin quality, as demonstrated in a structural equation model representing the contributions of skin color and skin texture. Other judgments related to health (facial attractiveness, facial symmetry, and structural aspects related to body weight) did not differ between the two groups. Perceptions of health were also responsible for differences in perceived spirituality, explaining folk hypotheses that Mormons are distinct because they appear more spiritual than non-Mormons.

**Conclusions/Significance:**

Subtle markers of group membership can influence how others are perceived and categorized. Perceptions of health from non-obvious and minimal cues distinguished individuals according to their religious group membership. These data illustrate how the non-conscious detection of very subtle differences in others' appearances supports cognitively complex judgments such as social categorization.

## Introduction

Whether passing briefly on the street, sitting opposite one another on a commuter train, or engaging in extensive conversation in a business meeting, we are consistently in the position of forming impressions of other people based on limited amounts of information. The ability to extract meaningful information about a person in our daily encounters may often be taken for granted. The rich cognitive complexity that forms the architecture for our capacity to perceive others often escapes our conscious awareness, emerging only as intuitive hunches or “gut feelings.” But although this sense of intuition leaves us with the feeling that our impressions of others are rough, ambiguous, and subjective, a growing body of evidence shows that our impressions of others can, in some cases, be fairly accurate. The current investigation elucidates one of these common hunches by providing empirical evidence for a surprising effect within interpersonal perception: the ability to accurately infer individuals' religious group membership from nothing more than their faces.

The human face is among the richest of all social stimuli in the human environment [1]. Not only does the face provide obvious cues to emotional expressions and intentions in its dynamic movements [2], even in its static form it can provide information about a variety of traits: personality attributes [3], individual identity [4], and group membership [5]. The last of these, group membership, is presently one of the best explored areas within which the face and its features can provide relevant and important information. We effortlessly and automatically extract information about individuals' sex and gender, race, and relative age from their faces [6]. Systematic investigations of the features involved in accurately perceiving an individual's gender, for example, have shown that multiple features convey information about whether a person is a man or a woman. Among these, several have been shown to be of key importance: the hair, the shape of the face, the eyes and brows, and even the mouth [7]–[8]. Similarly, although fewer empirical data have been presented for the facial cues that distinguish different racial groups, hair is known to be an important cue in some race judgments [9]–[11], and variation among race-distinctive features can have important effects upon individuals' perceptions [12]–[14].

Whereas the study of cues leading to the accurate perception of age, race and gender has led to important insights about the ability to perceive social information from nonverbal and appearance cues, the knowledge that can be gleaned from studying these categories may be limited by the general obviousness of the distinctions between the groups defined along those axes (e.g., men versus women for gender). The study of groups whose physical markers are less obvious may provide a unique window into the processes of social perception.

One fairly perceptually ambiguous social category is that of sexual orientation. Recent work has shown that the face provides information that allows individuals to judge others' sexual orientations with accuracy that is significantly higher than chance guessing [15]. These judgments can be accurately derived even when perceivers see the faces of gay and straight men and women for small fractions of a second [16]–[17]. Since this accuracy does not increase when perceivers are given more time to study the faces and because the brief perception of gay and straight faces has been shown to influence the processing of associated stereotypes, the perception of sexual orientation appears to occur automatically, just as is observed for the perception of age, race, and sex [17]–[18]. Subsequent study of the facial features that support the accuracy of these judgments has revealed that multiple cues express information about individuals' sexual orientations that can be used for accurate perception: the hair, eyes, and mouth; but these cues differ in the extent to which perceivers are aware of their use [19]. Perceivers appear to know that judgments of hairstyles allow them to accurately perceive others' sexual orientations but seem not to know that their judgments based on just the eyes or mouth also allow them to perceive sexual orientation accurately [19]. These data suggest that information about social group membership may be expressed simultaneously from multiple cues for use by both explicit and implicit perceptual processes—an insight that likely may not have been gained from studying groups for whom perception is obvious because the task of judging group membership from individual features might be too easy.

Similar to judgments of sexual orientation, there are other groups for whom individuals hold an intuitive sense of their ability to extract relevant information from nonverbal cues. One such intuition is the claim by some members of the Mormon religious faith (formally known as the Church of Jesus Christ of Latter Day Saints) that they can reliably discern who is Mormon and who is not Mormon from their appearance. In a recent study, we found evidence that this may be true [20]. Mormons and non-Mormons who passively observed the faces of both ingroup and outgroup members showed significantly better recognition memory for individuals belonging to their ingroup than they did for individuals belonging to their outgroup, similar to ingroup memory advantage effects commonly found for age [21], race [10]–[11], and gender [22]. Moreover, when perceivers were asked to explicitly indicate which of the faces they believed to be Mormon and non-Mormon, they were able to accurately categorize the individuals at rates significantly better than chance guessing. This was true for both Mormon and non-Mormon perceivers, living in both Mormon-populous and Mormon-scarce environments; but Mormons were more accurate than non-Mormons in making the distinction.

That Mormons and non-Mormons can be accurately categorized from their faces suggests that there must be a salient perceptual cue distinguishing the two groups. One explanation offered for the distinction between Mormons and non-Mormons is differences in expressed spirituality. For example, one Mormon woman described her experience with this phenomenon on her personal web-log with the following anecdote:

I ran into the TA whom I asked to speak on the Holy Ghost for my baptism. I was very excited to see him. There was this sense of “glow” from him, which I heard about many times yet never understood, like a “Mormon Radar.” But I saw it for the first time and I finally understood what it is. It is the Spirit! [23]

The belief that divine annunciation distinguishes Mormons from non-Mormons notwithstanding, other explanations may exist. For instance, Mormons are known to significantly differ from non-Mormons in their overall health, as measured by life expectancy [24]. Owing largely to their strict, substance-free lifestyle (which includes both illegal and legal recreational substances, such as alcohol, tobacco, and caffeine), Mormons are considered one of the healthiest populations of individuals in the United States [25]. Mormons show lower cancer rates [26]–[27] and overall lower mortality rates as compared to non-Mormons [28]. These differences are believed to be the effect of Mormons' substance-free lifestyle, routine health behaviors (i.e., exercise and well-balanced diet), early marriage, and regular church attendance [24], [29]. Indeed, a 24-year longitudinal study found Mormons to have a life expectancy rate 6–10 years longer than that of non-Mormon controls [29]. Early Church leaders, upon subjective observation of the Mormons' increased health advantage, also ascribed this distinction to divine influence:

The gift of the Holy Ghost … develops beauty of person, form and features. It tends to health, vigor, animation, and social feeling [30, p. 101].

Given that Mormons and non-Mormons do differ in their actual health and that individuals have subjectively reported the ability to read a difference between Mormons and non-Mormons from appearance cues, it seemed that the physical cue distinguishing Mormons and non-Mormons in their appearance may be visible signs of differences in health. We tested this hypothesis in the present work.

In light of recent research, it is not improbable that health could distinguish Mormons from non-Mormons. Jones et al., for instance, showed that judgments of health from high-resolution photos of patches of facial skin were related to perceivers' judgments of overall facial attractiveness [31]. Similarly, Roberts et al. found that perceptions of health from facial skin were related to individuals' actual health via genetic measures of immunological strength [32]. Thus, facial skin appears to carry valid cues to individuals' actual health [33]–[38]. Another facial cue associated with accurate judgments of individuals' health is their facial adiposity [39], [40]. Specifically, perceivers' judgments about an individual's apparent body weight from photos of only their faces significantly corresponded to their actual cardiovascular health, as measured by body-mass index, frequency and duration of respiratory illnesses, frequency of antibiotics use, and other measures of cardiovascular health [39]. In addition, some discussions of the components underlying differences in facial attractiveness have hypothesized that facial attractiveness advertises individuals' health [41], and that one key quality responsible for this is symmetry across the face's vertical axis [42]–[44].

If differences in health really are responsible for distinguishing Mormons' and non-Mormons' faces, it is likely that these cues are very subtle. Indeed, in the recognition memory studies reported above, participants expressed no awareness that the task concerned Mormons and non-Mormons [20]. This means that subtle differences in the faces of the two groups were likely extracted non-consciously in order to systematically affect the perceivers' later recognitions of the faces. Indeed, in that work we found that both environmentally- and experimentally-induced group salience worked to non-consciously prime perceivers to encode the faces according to religious group membership.

Elucidation of how perceivers are able to make these categorizations would therefore provide an interesting and informative illustration of the subtle manner by which we are able to incorporate basic perceptual cues into the cognitive-perceptual stream to support the construction of complex social judgments, such as social categorization. Moreover, that such a subtle distinction can exist and be perceived among an obscure set of targets would suggest that the cognitive machinery used for social categorization is quite flexible, permitting the application of basic perceptual distinctions to discriminating novel social groups as they are encountered by the cognitive system.

The present work therefore attempts to deconstruct the means by which perceivers extract information about Mormon and non-Mormon group membership from faces. In doing so, we first extend research demonstrating that individuals can make accurate judgments of Mormon/non-Mormon group membership from full faces in Study1 to show that participants are unaware of their accuracy in making these judgments. In Study 2, we then systematically test the physical features of the face that might potentially be involved in judging Mormon/non-Mormon group membership to determine which provide legible cues and which do not. Study 3 then specifically tests the hypotheses that the difference between Mormons' and non-Mormons' faces is based on perceptions of spirituality and health. Finally, Study 4 focuses specifically on the aspects of the face that have been previously found to express information about health and tests various statistical models to account for how these cues may lead to accurate judgments of Mormon/non-Mormon group membership.

## Methods

### Ethics

All of the studies reported in the current article were approved by the Institutional Review Board at Tufts University and all participants gave written informed consent and were treated in accordance with the ethical standards expressed in the Declaration of Helsinki. The photographs of persons used as stimuli for these studies were obtained from online personal advertisements within the Internet's public domain for which written permission for use was not required by the Institutional Review Board.

### Study 1

Perceivers can accurately distinguish between who is Mormon and non-Mormon based on facial appearance, but are they aware of the cues they use to make these judgments? To test this, we asked a group of individuals to categorize photos of the faces of male and female Mormon and non-Mormon adults and to estimate their accuracy on the task.

#### Stimuli

Stimuli were the same as those used in our earlier work on recognition memory for Mormon and non-Mormon faces [20]. Images of Mormon and non-Mormon men and women were obtained from online personal advertisements posted in various major cities across the United States. Search criteria were restricted to individuals 18–30 years of age who specifically indicated either active membership in the Church of Jesus Christ of Latter-day Saints or membership in another non-Mormon religious organization. Thus, all targets were explicitly Mormon or non-Mormon.

Research assistants who were blind to the hypotheses and intended use of the photos were assigned to gather either photos of Mormons or non-Mormons, so as to avoid any potential selection bias. Only photos of headshots were downloaded for use and only those images presenting a directly oriented face free of adornments (such as facial piercings or glasses) were selected for the experiment. Special attention was paid to variation in the faces according to the Mormon Church's appearance codes so that no obvious markers of Mormon or non-Mormon identity were present (e.g., women with more than one earring per ear would likely be non-Mormon and were excluded). Of the remaining photos, we randomly selected photos of 40 Mormon/non-Mormon men and 40 Mormon/non-Mormon women for a total of 160 photos (80 Mormon, 80 non-Mormon) using a random-number generator. All of the targets were Caucasian.

**Figure 1:**
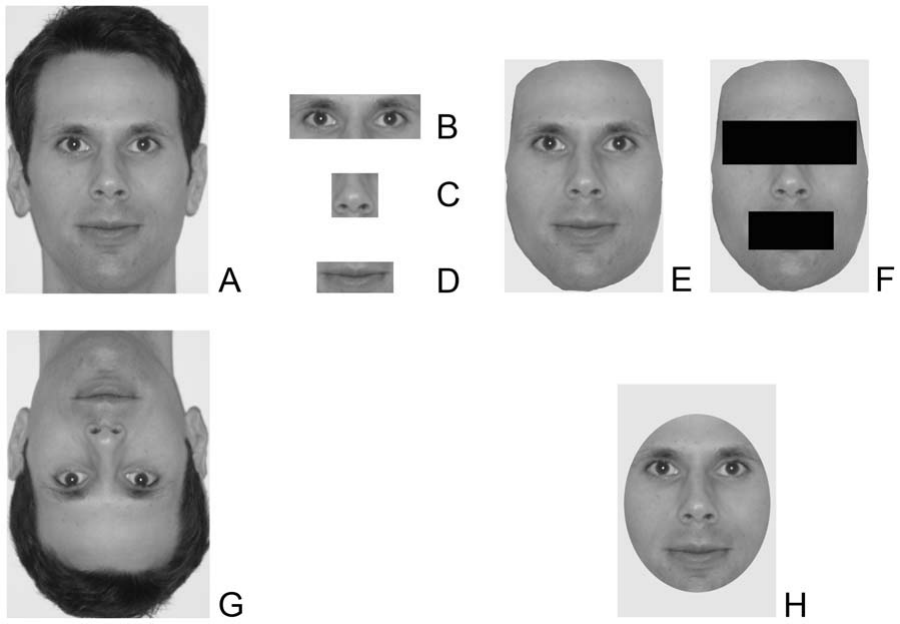
Sample stimuli prepared in the same manner as those used in each of the conditions across Studies 1–4. (a) unaltered but standardized photo from Studies 1, 3, and 4; (b) eyes/brows only condition from Study 2; (c) nose only condition from Study 2; (d) mouth only condition from Study 2; (e) hairless face condition from Study 2; (f) hairless face with eyes and mouth occluded from Study 2; (g) inverted face condition from Study 2; (h) outer shape removed condition from Study 2.

The photos were cropped to the smallest frame that included the sides and tops of targets' hair and the bottom of their chin. Thus, neck jewelry, clothing, and image backgrounds were not visible. The photos were then converted to grayscale and standardized for size. Four naïve research assistants (Cronbach's α = .83) rated each face for affective expression from 1 (*Neutral*) to 4 (*Happy*) to 7 (*Very Happy*), which showed no significant differences between the two groups: *t*(158) = 0.04, *p* = .97; none of the targets expressed emotions that did not fall along the spectrum between neutral and happy (e.g., disgust, fear, sadness, anger, surprise, or contempt). A sample photo is presented in Figure 1A.

#### Procedure

Twenty-three undergraduates (*n = *19 females) categorized each of the faces as Mormon or non-Mormon in exchange for partial course credit; none of the participants was Mormon for any of the studies reported in this work. Participants were presented with each face in random order on a computer screen and instructed to categorize each target according to his or her probable group membership via key-press. Participants were encouraged to work quickly and to base their judgments on their first impressions. After completing the task, participants were asked to estimate the percentage of faces that they believed they had accurately categorized from 0–100%.

### Study 2

In Study 1, we observed that participants could accurately judge who was Mormon and non-Mormon from photographs of faces, despite a relative lack of awareness of their ability to do so. Here we tested various facial features to determine which might permit accurate judgments in order to gain an understanding of what cues perceivers are using in making their judgments.

Each photo from Study 1 was cropped to show only the targets' eyes/brows; nose; lips/mouth; face without hair; face without hair, eyes, or mouth; face inverted; and face with outer-shape removed (see Figures 1B–1H). 146 undergraduates (*n = *92 females) were randomly assigned to categorize the stimuli from one of these conditions in exchange for partial course credit. Instructions and procedures were the same as in Study 1.

### Study 3

Study 2 uncovered that many of the facial features found most critical for judgments of other social groups, such as eyes [7]–[8], hairstyles [11], [19], and mouths [10], did not provide sufficient information for distinguishing Mormons from non-Mormons. Rather, the features remaining in the most minimal condition for which Mormons and non-Mormons could be distinguished primarily left information about the skin and facial structure visible. Both skin [31]–[38] and facial structure, via adiposity [39], [40], have been found important in the accurate perception of individuals' health. Given that Mormons and non-Mormons are known to significantly differ in their levels of health, it therefore seemed possible that differences in health may serve as the basis for perceivers' Mormon/non-Mormon categorizations. Yet folk hypotheses have alternatively suggested that spirituality may be the distinguishing factor between Mormons and non-Mormons. In Study 3, we therefore asked separate groups of participants to rate the Mormon and non-Mormon faces for their levels of health and spirituality.

Fifty-four undergraduates (*n = *37 females) were randomly assigned to rate each target from Study 1 along a scale anchored at 1 (*Not at all spiritual*) and 7 (*Very spiritual*; *n = *25, Cronbach's α = .74) or 1 (*Not at all healthy*) and 7 (*Very healthy*; *n = *29, Cronbach's α = .94). Each photo was presented by computer in random order.

### Study 4

The results of Study 3 suggested that health may be the primary cue distinguishing Mormons from non-Mormons and that it may even account for the folk belief that Mormons and non-Mormons differ in spirituality. Numerous studies, however, have shown that health can be accurately judged from numerous facial features, including skin color [33]–[37], skin texture [38], facial structure [39], [40], facial attractiveness [31]–[32], [36]–[38], [41]–[42], [44], and facial symmetry [40]–[43]. In Study 4 we therefore investigated which of these features might contribute to the relationship between targets' perceived health and their Mormon/non-Mormon group membership.

Eighty participants (*n* = 44 females) rated each target from Study 1 on attractiveness, facial symmetry, or skin texture. Participants made their judgments along 7-point scales, respective to their conditions: 1 (*Not at all attractive*) to 7 (*Very attractive*; *n = *20, Cronbach's α = .93), 1 (*Very asymmetrical*) to 7 (*Very symmetrical*; *n* = 30; Cronbach's α = .85), or 1 (*Smooth skin*) to 7 (*Rough skin*; *n* = 30, Cronbach's α = .75). Each photo was presented by computer in random order.

### Study 5

If Mormons and non-Mormons are distinguished based on perceptions of health from their faces, naïve non-Mormon perceivers (such as those participating in these studies) must possess some knowledge that Mormons and non-Mormons differ in health. Study 5 tested this by asking a random sample of non-Mormon respondents from the same participant population to rate the typical health of members of a series of social groups that included Mormons.

We constructed a survey in which participants were asked to rate the expected health of a typical member from each of a series of social groups along the same 7-point health scale as in Study 4A. The two critical groups of interest in the survey were Mormons and Protestants, the latter serving as the default non-Mormon outgroup in the United States. Fifty-five undergraduates completed the survey (Cronbach's α = .97).

## Results

### Study 1

As in previous studies on the accurate categorization of group membership [15]–[17], [20], [45], data were analyzed using signal detection with Mormons arbitrarily treated as signal and non-Mormons arbitrarily treated as noise. Thus, the proportion of Mormon targets that each participant categorized as Mormon and the proportion of non-Mormon targets that each participant categorized as Mormon constituted the hit and false-alarm rates, respectively (see Table 1 for descriptive statistics). As in previous work [20], participants' accuracy (*A*') in categorizing the faces was significantly better than the chance guessing rate of .50 [*t*(22) = 3.37, *p* = .003, *r* = .58] while measures of response bias (*B*') [45] showed that perceivers categorized more targets as non-Mormon than Mormon. Although the mean rate of accuracy observed here was relatively low, it is on par with the effects reported for other nonverbal facial cues, such as rates of accuracy in categorizing certain facial expressions of emotion [46]. Seventy-four percent of participants categorized the targets at rates better than chance guessing and there were no significant differences in accuracy between male and female participants in this study [*t*(21) = 1.00, *p* = .33] or in any of those that follow; we therefore consider participant gender no further.

**Table 1:** Summary statistics for the signal detection analyses in Studies 1–2.

			Hits		False-Alarms		Accuracy (*A*')		Response Bias (*B*')	
	Condition	*n*	*M*	*SD*	*M*	*SD*	*M*	*SD*	*M*	*SD*
Study 1	Full photos	19	.36	.12	.30	.13	.56	.09	.06	.08
Study 2	Eyes/brows only	25	.38	.14	.38	.15	.51	.09	.02	.12
	Noses only	20	.33	.12	.32	.13	.52	.09	.04	.17
	Mouths only	22	.35	.15	.35	.16	.51	.08	.02	.10
	Hairless faces	19	.35	.10	.31	.09	.54	.06	.03	.05
	No hair, eyes/brows, or mouth	20	.37	.15	.34	.14	.54	.07	.03	.08
	Inverted faces	20	.35	.13	.33	.14	.54	.07	.04	.09
	Outer shape removed	20	.43	.12	.39	.11	.54	.07	.02	.08

Similar to previous work on the categorization of male sexual orientation from full faces, participants' estimated accuracy was not correlated with their actual accuracy in judging the faces: *r*(21) = −.07, *p* = .76. Indeed, participants provided rather low estimations for their accuracy: *M* = 28%, *SD*  = 17%. These scores were consistent with the participants' self-reports during debriefing, in which the vast majority reported that they had been guessing throughout the task. Thus, perceivers do not appear to possess much conscious awareness of their ability to extract information about Mormon/non-Mormon group membership from photos of faces, yet they are more accurate than chance in doing so. In Study 2, we therefore explored the features that might be involved in these judgments.

### Study 2

Participants' accuracy was no better than chance for their categorizations of the eyes/brows [*t*(24)  = 0.64, *p* = .53], noses [*t*(19) = 0.98, *p* = .34], and mouths [*t*(21) = 0.37, *p* = .71]. However, participants' accuracy was significantly better than chance for their categorizations of the faces without hair [*t*(18) = 2.74, *p* = .01, *r* = .54]; without hair, eyes, or mouth [*t*(19) = 2.38, *p* = .03, *r* = .48]; inverted faces [*t*(19) = 2.48, *p* = .02, *r* = .49]; and with outer shape removed [*t*(19) = 2.26, *p* = .03, *r* = .46]. Comparison of these conditions against the full photos in Study 1 showed no significant differences in accuracy: all *t*'s <.94, all *p*'s >.35. Response bias scores showed that participants uniformly categorized more faces as non-Mormon than Mormon.

Similar to Study 1, although participants could accurately discern Mormon/non-Mormon group membership from some of the features, their estimations did not reflect their actual accuracy (*M* = 26%, *SD*  = 19%); all *r*'s <.15, all *p*'s >.24. Thus, perceivers seemed relatively unaware of the basis for their judgments, hinting at the subtlety of the perceptual cues involved and suggesting that the information was being processed non-consciously.

The present data suggest that eyes/brows, mouths, and noses do not carry sufficient signal to distinguish Mormons from non-Mormons. In contrast, when hairstyles, eyes, mouths, and outer face shape were removed, participants' categorizations were statistically equivalent to their judgments of the full photos in Study 1. Similarly, inverting the faces to disrupt configural processing [47] did not impair perceivers' accuracy. This process of elimination leaves very few common features that could be responsible for the participants' accuracy, but several of those remaining have shown relationships to perceptions of health from faces. Global facial symmetry along the vertical axis would not have been affected by our manipulations, nor would skin health, facial attractiveness, and many structural cues to facial adiposity. Given that each of these cues is relatively subtle (outside of great extremes), it seems tenable that one or more might be responsible for the distinction between Mormon and non-Mormon faces. Studies 3 and 4 therefore tested this hypothesis in more detail.

### Study 3

Consistent with the folk belief that Mormons and non-Mormons differ in expressions of spirituality, Mormons (*M* = 4.11, *SE*  = .08) were rated as significantly more spiritual than non-Mormons (*M* = 3.26, *SE*  = .23): *t*(158) = 2.72, *p* = .007, *r* = .21. Consistent with epidemiological research showing that Mormons and non-Mormons differ in health, Mormons (*M* = 4.83, *SE*  = .08) were also rated as significantly healthier than non-Mormons (*M* = 4.59, *SE*  = .09): *t*(158) = 2.05, *p* = .04, *r* = .16.

**Figure 2:**
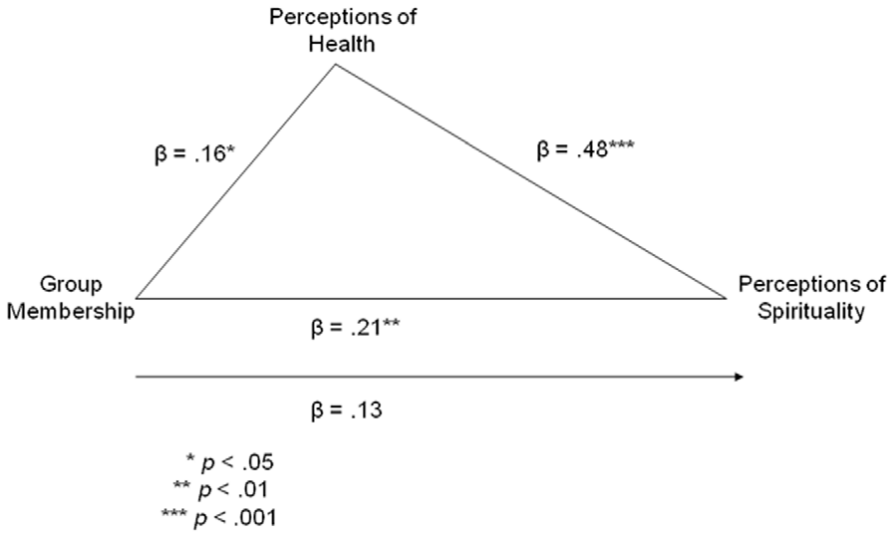
Statistical model illustrating the role of health in mediating the relationship between Mormon/non-Mormon group membership and perceptions of spirituality; standardized coefficients (β's) are provided neighboring the model's paths with indications of statistical significance.

As Mormons are distinct from non-Mormons in their health behaviors [24]–[29], we wondered whether Mormons' folk belief about the detectability of Mormon identity from appearance may be due to differences in apparent health. We therefore conducted a mediation analysis [48] of the relationship between targets' group membership and spirituality ratings, employing health ratings as the mediating variable. We dummy-coded targets' group membership of non-Mormon and Mormon as 0 and 1, respectively, and entered this as the predictor variable in an OLS regression mediation model predicting ratings of spirituality. As displayed in the illustrated model in Figure 2, the paths between all three variables were significant and the direct path became non-significant under the influence of the mediator. These data suggest the presence of at least partial mediation, which was confirmed by a statistically significant Sobel test score: *Z* = 1.99, *p*<.05. Thus, the relationship between perceivers' ratings of the spirituality of Mormon and non-Mormon faces and the targets' memberships in those groups appears to be mediated by perceptions of health from the faces.

### Study 4

Data were analyzed by averaging across the participants' ratings for each face such that targets were the unit of analysis. Comparisons of the ratings for the Mormon and non-Mormon targets showed no significant differences in terms of attractiveness [*M*_Mormon_  = 3.61, *SE*  = .10, *M*_non-Mormon_  = 3.44, *SE*  = .09; *t*(158) = 1.25, *p* = .21], symmetry [*M*_Mormon_  = 4.35, *SE*  = .07, *M*_non-Mormon_  = 4.21, *SE*  = .07; *t*(158) = 1.40, *p* = .17], or skin texture [*M*_Mormon_  = 3.62, *SE*  = .05, *M*_non-Mormon_  = 3.69, *SE*  = .05; *t*(158)  = 0.91, *p* = .36]. To assess skin color and facial structure, we measured the pixel values and dimensions of the facial features, respectively.

To measure skin color, we averaged the grayscale values across the portions of the face revealed by the eyes- and mouth-covered condition of Study 2 (Figure 1F) for each face. Comparison of the Mormon and non-Mormon targets' skin tone showed no significant difference between the two groups [*M*_Mormon_  = 29.19, *SE*  = .88, *M*_non-Mormon_  = 27.46, *SE*  = .81; *t*(158) = 1.44, *p* = .15].

To measure the targets' weight as expressed by their faces, we measured the distances across the cheekbones, from the upper eyelid to the lips, and across the jaw through the center of the mouth. These distances were used to construct ratios of cheek-width to face-height and cheek-width to jaw-width, following the procedures for the accurate measure of facial adiposity provided by earlier work [40]. Neither the ratio for cheek-width to face-height [*M*_Mormon_  = 1.12, *SD*  = .08, *M*_non-Mormon_  = 1.13, *SD*  = .09; *t*(158)  = 0.95, *p* = .34] nor cheek-width to jaw-width [*M*_Mormon_  = 1.93, *SD*  = .29, *M*_non-Mormon_  = 2.00, *SD*  = .41; *t*(158) = 1.18, *p* = .24] significantly differed between the Mormon and non-Mormon targets.

**Figure 3:**
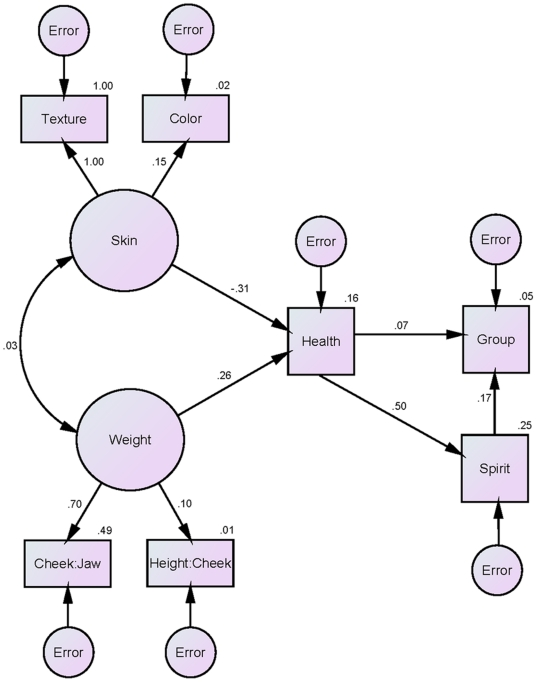
Structural equation model testing the contributions of factors related to skin quality and weight to predicting targets' Mormon and non-Mormon group membership and perceptions of spirituality from their faces, via perceptions of health. Path coefficients are standardized (β's), error variances for the endogenous variables are indicated in ovals, and variances explained (multiple *R* ^2^ values) are adjacent to the endogenous variables.

As none of these variables showed significant differences between the Mormon and non-Mormon targets, we reasoned that their cumulative partial contributions may be responsible for the relationship between targets' group membership and their perceptions of health. We therefore fit a structural equation model using AMOS to test the hypothesis that latent constructs representing the dimensions of health related to skin (texture and color) and weight (cheek-width to jaw-width and cheek-width to height) would predict participants' ratings of health from the faces, which in turn would predict both ratings of spirituality and group membership (see Figure 3). The initial model fitting these dimensions showed a relatively poor overall fit and a negative variance related to the texture variable resulted in a standardized path co-efficient exceeding 1.00. To correct for this Heywood case [49], we constrained the variance to 0 [50]. The chi-square test measuring the difference between the estimated values for the paths against the observed values for the paths was significant [χ^2^ (12, *N* = 160) = 38.23, *p*<.001], the comparative fit index (CFI  = .73) did not meet the .95 criterion needed for goodness of fit, and the root mean-square error of approximation (RMSEA  = .12) exceeded the .08 threshold, indicating a poor fit of the model as well.

**Figure 4:**
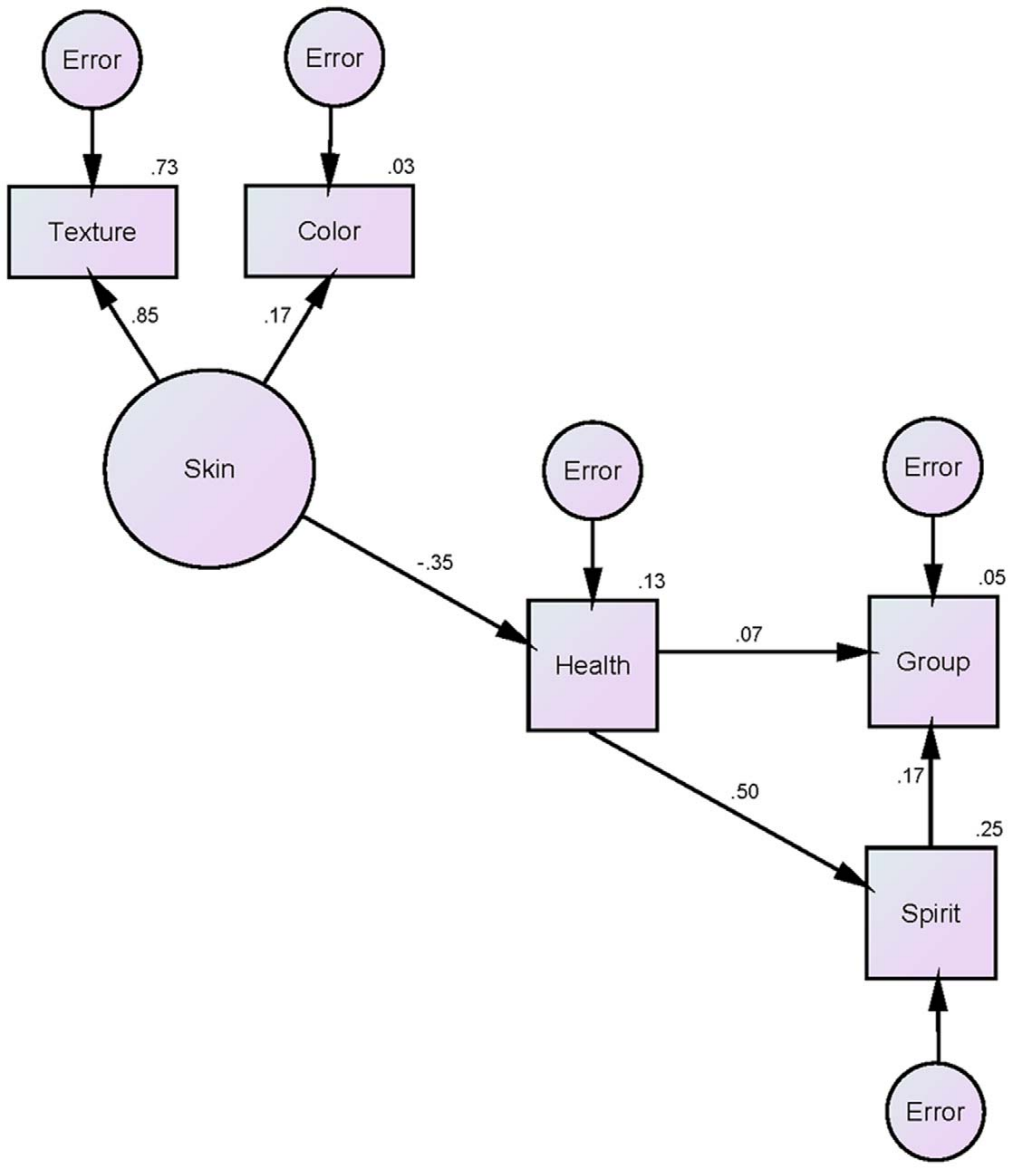
Trimmed structural equation model in which variables related to skin quality are tested as predictors of targets' Mormon/non-Mormon group membership and perceived spirituality by way of perceived health. Path coefficients are standardized (β's), error variances for the endogenous variables are indicated in ovals, and variances explained (multiple *R* ^2^ values) are adjacent to the endogenous variables.

We therefore trimmed the model, separating the skin and weight latent constructs as independent predictors. The model in which weight and its constituent factors predicted target group membership and spirituality ratings by way of health initially failed to estimate due to another Heywood case respective to a negative error variance estimate for the cheek-width to jaw-width variable. Constraining the parameter to 0, however [49], allowed the model to be estimated but showed a poor overall fit: χ^2^ (5, *N* = 160) = 25.47, *p*<.001; CFI  = .73; RMSEA  = .16. However, the model in which skin and its constituent factors predicted group membership and spirituality via health resulted entirely in positive variances and showed a good overall fit: χ^2^ (4, *N* = 160) = 5.24, *p* = .26; CFI  = .98; RMSEA  = .04 (see Figure 4). Moreover, this model was a significantly better fit to the data than both the initial model that included weight and skin [χ^2^ (8) = 32.99, *p*<.001] as well as the trimmed model that included only weight [χ^2^ (1) = 20.23, *p*<.001]. We therefore reasoned that skin color and texture provide an important contribution for perceiving health from the Mormon and non-Mormon targets' faces, allowing for reliable perceptions of spirituality from the faces and accurate prediction of group membership.

### Study 5

Consistent with other participants' naïve perceptions of the Mormon and non-Mormon faces, respondents to the survey believed the typical Mormon (*M* = 4.58, *SD*  =  .93) to be significantly healthier than the typical Protestant (*M* = 4.18, *SD*  = .69): *t*(54)  = 3.86, *p*<.001, *r* = .47. Thus, even if participants do not possess conscious awareness of their ability to distinguish Mormons from non-Mormons, as observed in Studies 1 and 2, they do hold explicit knowledge that Mormons are typically healthier than non-Mormons and appear to apply this knowledge—even if only non-consciously—in making their categorizations of individuals as Mormon and non-Mormon.

## Discussion

People are adept at perceiving others' group memberships. Indeed, here we found that people could distinguish between members of two perceptually ambiguous groups—Mormons and non-Mormons—from subtle differences in their facial appearance. But although perceivers could categorize the Mormons and non-Mormons significantly more accurately than chance, they appeared relatively unaware of their ability to do so. Information about health from the faces seemed to form the basis for perceivers' categorizations of Mormon/non-Mormon group membership, with facial skin quality serving as the primary cue distinguishing the two groups. Given the subtle, partial, and cumulative expression of health from the skin, perceivers seemed relatively unaware that they were utilizing information about health in making their judgments—despite possessing explicit knowledge that the two groups differ in their actual health. These data therefore speak to individuals' capacity for incorporating information about others' social group memberships from very subtle and minimal visual cues.

Study 1 showed that perceivers were capable of judging who was Mormon and non-Mormon based only on photographs of faces but that their estimations of accuracy did not match their actual ability to make the categorizations. Study 2 then established that none of the eyes, the nose, nor the mouth carried sufficient signal to differentiate between the groups. However, accuracy was unimpaired when faces were inverted to disrupt holistic processing, when targets' hairstyles or outer face shape were removed, or when hairstyle was removed while blocking out the eyes/brows and mouth; and perceivers remained unaware of their ability to make accurate judgments from these cues. Studies 3 and 4 showed that the differences between Mormons' and non-Mormons' faces were related to perceptions of health. Study 3 showed that Mormons and non-Mormons significantly differed both in perceived health and perceived spirituality but that perceptions of spirituality were based upon perceptions of health. Study 4 elucidated this relationship by testing a series of structural equation models that incorporated multiple known cues to facial health, extending the model in Study 4 to demonstrate that the primary contributor to the group health differences is facial skin quality. Finally, Study 5 surveyed individuals from the same population of naïve non-Mormon participants to show that they possess the knowledge of differences in health between Mormons and non-Mormons that would be needed to use health, even if non-consciously, as a cue to judge Mormon/non-Mormon group membership.

These data provide novel insights regarding how social groups are categorized and perceived. Because the features distinguishing members of many groups (such as age, race, and sex) are so obvious, little is known about the human capacity to perceive and categorize less obvious group members. Previous research has documented that people can discriminate between members of perceptually ambiguous groups, such as gay versus straight [15]–[19]. Yet despite advances in understanding aspects of the perceivers' and targets' roles in these judgments [15], it has remained somewhat mysterious as to how these groups actually differ. The current data indicate that very subtle differences signaling group membership permit the accurate discrimination of nonobvious social groups, suggesting that the perceptual system makes use of subtle cues to support higher-order cognitive and behaviorally consequential outcomes, such as accurately judging another's category membership.

This work has some limitations that may provide opportunities for future research. First, we were limited in that we only tested these perceptions among non-Mormons living in an environment with very few Mormons. As our participants expressed very low levels of exposure to Mormons or Mormon culture, it is very interesting that they were still capable of distinguishing Mormons from non-Mormons. Because Mormons have been found to be more accurate in categorizing members of the two groups [20], it is possible that the present data provide more conservative estimates of the strength of these effects. It would therefore be interesting to investigate these same effects among Mormon perceivers. Moreover, although participants possessed knowledge that Mormons are generally healthier than non-Mormon Protestants, it would be interesting to test the generality of the relationship between spirituality, health and categorization in other groups and also to examine whether another set of cues and features might be relevant for other groups. Finally, although the present data show that Mormons and non-Mormons may be distinguished by cues to health in their faces, the present data say nothing about the actual health of our Mormon and non-Mormon targets. Although previous work in the medical and epidemiological literatures has shown that Mormons are significantly healthier and live longer than non-Mormon controls [24]–[29], the present findings add to this literature by showing that these cues to health are advertised in the targets' faces and are utilized for social categorization.

In conclusion, Mormons and non-Mormons subtly differ in their facial appearance and perceivers are able to perceive these differences in a way that allows for accurate categorization. The two groups are distinguished by differences in apparent health, which appears to be expressed in facial cues signaling skin quality. These data verify a longstanding folk belief among a highly cohesive minority religious group and provide insights to the incorporation of subtle perceptual cues to support higher-level social cognitions.

## References

[1] Zebrowitz LA (1997). Reading faces: Window to the soul?.

[2] Ambady N, Bernieri FJ, Richeson JA (2000). Toward a histology of social behavior: Judgmental accuracy from thin slices of the behavioral stream.. Adv Exp Soc Psychol.

[3] Rule NO, Ambady N, Adams RB (2009). Personality in perspective: Judgmental consistency across orientations of the face.. Perception.

[4] Macrae CN, Quinn KA, Mason MF, Quadflieg S (2005). Understanding others: The face and person construal.. J Pers Soc Psychol.

[5] Brebner JL, Martin D, Macrae CN (2009). Dude looks like a lady: Exploring the malleability of person categorization.. Euro J Soc Psychol.

[6] Macrae CN, Bodenhausen GV (2000). Social cognition: Thinking categorically about others.. Ann Rev Psychol.

[7] Brown E, Perrett DI (1993). What gives a face its gender?. Perception.

[8] Roberts T, Bruce V (1988). Feature saliency in judging the sex and familiarity of faces.. Perception.

[9] Ellis HD, Deregowski JB, Shepherd JW (1975). Descriptions of White and Black faces by White and Black subjects.. Intl J Psychol.

[10] MacLin OH, Malpass RS (2001). Racial categorization of faces: The ambiguous race face effect.. Psychol Pub Pol Law.

[11] MacLin OH, Malpass RS (2003). The ambiguous-race face illusion.. Perception.

[12] Blair IV, Judd CM, Sadler MS, Jenkins C (2002). The role of Afrocentric features in person perception: Judging by features and categories.. J Pers Soc Psychol.

[13] Maddox KB (2004). Perspectives on racial phenotypicality bias.. Pers Soc Psychol Rev.

[14] Eberhardt JL, Davies PG, Purdie-Vaughns VJ, Johnson SL (2005). Looking deathworthy: Perceived stereotypicality of Black defendants predicts capital-sentencing outcomes.. Psychol Sci.

[15] Rule NO, Ambady N, Adams RB, Macrae CN (2007). Us and them: Memory advantages in perceptually ambiguous groups.. Psycon Bull Rev.

[16] Rule NO, Ambady N (2008). Brief exposures: Male sexual orientation is accurately perceived at 50 ms.. J Exp Soc Psychol.

[17] Rule NO, Ambady N, Hallett KC (2009). Female sexual orientation is perceived accurately, rapidly, and automatically from the face and its features.. J Exp Soc Psychol.

[18] Rule NO, Macrae CN, Ambady N (2009). Ambiguous group membership is extracted automatically from faces.. Psychol Sci.

[19] Rule NO, Ambady N, Adams RB, Macrae CN (2008). Accuracy and awareness in the perception and categorization of male sexual orientation.. J Pers Soc Psychol.

[20] Rule NO, Garrett JV, Ambady N (2010). Faces and places: Geographic environment influences the ingroup memory advantage.. J Pers Soc Psychol.

[21] Wright DB, Stroud JN (2002). Age differences in lineup identification accuracy: People are better with their own age.. Law Hum Behav.

[22] Wright DB, Sladden B (2003). An own gender bias and the importance of hair in face recognition.. Acta Psychol.

[23] Nauvoo (2008). Web-log.. http://nauvoo.wordpress.com/.

[24] Merrill RM (2004). Life expectancy among LDS and non-LDS in Utah.. Demographic Res.

[25] Manton KG, Stallard E, Tolley HD (1991). Limits to human life expectancy: Evidence, prospects, and implications.. Popul Devel Rev.

[26] Enstrom JE (1978). Cancer and total mortality among active Mormons.. Cancer.

[27] Lyon JL, Gardner K, Gress RE (1994). Cancer incidence among Mormons and Non-Mormons in Utah (United States) 1971-85.. Canc Caus Cont.

[28] Enstrom JE (1989). Health practices and cancer mortality among active California Mormons.. J Natl Canc Inst.

[29] Enstrom JE, Breslow L (2008). Lifestyle and reduced mortality among active Mormons, 1980-2004.. Prevent Med.

[30] Pratt PP (1855). Key to the Science of Theology, 9th ed..

[31] Jones BC, Little AC, Burt DM, Perrett DI (2004). When facial attractiveness is only skin deep.. Perception.

[32] Roberts SC, Little AC, Gosling LM, Perrett DI, Carter V (2005). MHC-heterozygosity and human facial attractiveness.. Evol Hum Behav.

[33] Stephen ID, Coetzee V, Law Smith MJ, Perrett DI (2009). Skin blood perfusion and oxygenation color affect perceived human health.. PLoS ONE.

[34] Stephen ID, Law Smith MJ, Stirrat MR, Perrett DI (2009). Facial skin coloration affects perceived health of human faces.. Intl J Primatol.

[35] Fink B, Matts PJ (2008). The effects of skin colour distribution and topography cues on the perception of female facial age and health.. J Euro Acad Derm Vener.

[36] Matts PJ, Fink B, Grammer K, Burquest M (2007). Color homogeneity and visual perception of age, health, and attractiveness of female facial skin.. J Am Acad Dermatol.

[37] Fink B, Grammer K, Matts PJ (2006). Visible skin color distribution plays a role in the perception of age, attractiveness, and health in female faces.. Evol Hum Behav.

[38] Fink B, Grammer K, Thornhill R (2001). Human (*Homo sapiens*) facial attractiveness in relation to skin texture and colour.. J Comp Psychol.

[39] Coetzee V, Perrett DI, Stephen ID (2009). Facial adiposity: A cue to health?. Perception.

[40] Coetzee V, Chen J, Perrett DI, Stephen ID (2010). Deciphering faces: Quantifiable visual cues to weight.. Perception.

[41] Rhodes G (2006). The evolution of facial attractiveness.. Ann Rev Psychol.

[42] Thornhill R, Gangestad SW (1993). Human facial beauty: Averageness, symmetry, and parasite resistance.. Hum Nat.

[43] Jones BC, Little AC, Feinberg DR, Penton-Voak IS, Tiddeman BP (2004). The relationship between shape symmetry and perceived skin condition in male facial attractiveness.. Evol Hum Behav.

[44] Rhodes G, Yoshikawa S, Palermo R, Simmons LW, Peters M (2007). Perceived health contributes to the attractiveness of facial symmetry, averageness, and sexual dimorphism.. Perception.

[45] Quanty MB, Keats JA, Harkins SG (1975). Prejudice and criteria for identification of ethnic photographs.. J Pers Soc Psychol.

[46] Elfenbein HA, Ambady N (2002). Is there an in-group advantage in emotion recognition?. Psychol Bull.

[47] Santos IM, Young AW (2008). Effects of inversion and negation on social inferences from faces.. Perception.

[48] Baron RM, Kenny DA (1986). The moderator-mediator variable distinction in social psychological research: Conceptual, strategic, and statistical considerations.. J Pers Soc Psychol.

[49] Kline RB (1998). Principles and practices of structural equation modeling..

[50] Cortina JM (2002). Big things have small beginnings: An assortment of “minor” methodological misunderstandings.. J Manag.

